# Dose–response relationship between active smoking and lung cancer mortality/prevalence in the Chinese population: a meta-analysis

**DOI:** 10.1186/s12889-023-15529-7

**Published:** 2023-04-24

**Authors:** Feiling Ai, Jian Zhao, Wenyi Yang, Xia Wan

**Affiliations:** Institute of Basic Medical Sciences, Chinese Academy of Medical Sciences, School of Basic Medicine, Peking Union Medical College, Beijing, 100005 China

**Keywords:** Smoking, Lung cancer, Dose‒response relationship, Chinese

## Abstract

**Background:**

The dose‒response relationship-based relative risk (RR) of smoking exposure could better predict the risk of lung cancer than the dichotomous RR. To date, there is a lack of large-scale representative studies illustrating the dose‒response relationship between smoking exposure and lung cancer deaths, and no study has systematically pooled the current evidence in the Chinese population.

**Objectives:**

To elucidate the dose‒response relationship of smoking and the risk of lung cancer mortality in the Chinese population.

**Methods:**

Data were derived from studies on dose‒response relationships of smoking exposure and the risk of lung cancer among Chinese adults published before June 30^th^, 2021. Based on smoking exposure indicators and RR of lung cancer mortality, a series of dose‒response relationship models were developed. For smokers, 10 models were built to fit the dose‒response relationships between pack-years and RR of lung cancer deaths. For quitters, quit-years and corresponding RRs were used, and the pooled dichotomous RR value was used as the starting point to avoid overestimation. Finally, the results were compared with the estimates from 2019 Global Burden of Disease (GBD) study.

**Results:**

A total of 12 studies were included. Among 10 dose‒response relationship models of pack-years with the RR of lung cancer mortality, the integrated-exposure–response (IER) model achieved the best fit. In all models, less than 60 pack-years presented RRs below 10. For former smokers, the RR decreased to 1 when quit-years reached up to 7 years. Both smokers and quitters had much lower RRs than that of the global level estimated by GBD.

**Conclusion:**

The risk of lung cancer mortality rose with pack-years and decreased with quit-years among Chinese adults, and both values were far below global level. The results suggested that the dose–response RR of lung cancer deaths associated with smoking in China should be estimated separately.

**Supplementary Information:**

The online version contains supplementary material available at 10.1186/s12889-023-15529-7.

## Introduction

Smoking is the most significant risk factor for lung cancer, and over 80% of male lung cancer deaths can be attributed to smoking [1]. Over the past 30 years, the tobacco epidemic has persisted and the pattern of smoking is relatively stable in China, with prevalence higher than 50% among males aged 15 years and above [2]. China has a large number of smokers, up to 300 million in 2018 [3]. Moreover, the mortality rate of lung cancer is still on the rise in China, which ranks first in both morbidity and mortality rates from malignant tumors [4]. In 2015, there were approximately 730,000 diagnosed patients and 610,000 deaths due to lung cancer in China [5]. There is a lag effect of smoking exposure on cancer, and although smoking prevalence in the general Chinese population has declined over the past three decades, a study predicted that smoking-related mortality would continue to surge in the next 20 years [6].

The high exposure to cooking oil fumes and biomass fuels has competing effects on the Chinese population due to burning coal for domestic heating and the unique cooking style in China. Therefore, the relative risks (RRs) of smoking-attributable diseases were significantly different in China compared with those in developed countries [7–9]. It is well established that the RR of lung cancer from smoking was stable (2 ~ 5) in the Chinese population in the 1980s [10, 11], and it was significantly lower than the RRs estimated in Europe and the United States [12, 13]. For example, in the United States, the RRs for lung cancer in 1959–1965, 1982–1988 and 2000‒2010 were 12.22, 23.81 and 24.97 for males, and 2.73, 12.65 and 25.66 for females, respectively [14]. Therefore, the Global Burden of Disease (GBD) study estimated the RRs of smoking and attributable diseases in China separately before 2017.

In 2000‒2010, GBD used the estimated values from a case–control study conducted by Liu et al. [10] in a population of 1 million in 24 urban and 73 rural areas in China; the RR of lung cancer among Chinese male smokers and female smokers was 2.72 and 2.64, respectively. In 2013, the results of the China Kadoorie Biobank (CKB) (2006–2011), a prospective study of chronic diseases in China conducted in 5 urban and 5 rural areas among 500,000 people in China, were used [15]. In 2015, the follow-up of CKB study was updated to 2014, and the RRs of lung cancer among male and female smokers were 2.58 and 2.56, respectively [11].

Smoking status could not present the cumulative effects of smoking because frequency and volume varied among smokers, therefore, researchers further designed continuous variables to demonstrate the intensity of smoking. The GBD research further proposed a new method to utilize the dose‒response relationship of smoking with mortality from lung cancer in 2017 [16, 17]. They pooled global studies and estimated global harmonized RR dose‒response relationship models of smoking-attributable diseases, classifying individuals into nonsmokers, current smokers and former smokers [18]. Notably, in the updated method, different sexes and regions used the same RR values. The GBD 2017 showed that the RR of lung cancer death increased from 1.76 to 21.52 when pack-years increased from 5 to 85.7 [16]. Similarly, the 2019 study showed that RR increased from 3.43 to 20.9 when pack-years of smoking increased from 10 to 100 [19].

It has previously been suggested that the dichotomous RRs of lung cancer caused by smoking are significantly different in China from those in developed countries. By analogy to the dose‒response relationship RRs, we supposed that there might also be more significant differences. Therefore, it is necessary to summarize the latest evidence and perform dose‒response relationship study to estimate the RR of lung cancer deaths attributable to smoking in China, and compare the results with the global level calculated by GBD, so that we can estimate the burden of smoking-induced diseases more reasonably.

## Methods

### Data source

The study was based on the meta-analysis database of smoking and related diseases in the Chinese population published elsewhere [20, 21]. The PubMed, Embase, Cochrane, CNKI, WanFang and VIP databases were searched. All publicly published cohort and case‒control studies of smoking and related diseases in the Chinese population from the database establishment to June 30^th^, 2021, were collected, including Chinese and English language studies. The included studies were (1) original research and full text available; (2) conducted in Chinese populations with representativeness; (3) case‒control or cohort studies (prospective, retrospective cohort studies and nested case‒control studies); and (4) reported odds ratios (OR), or RR, hazard ratios (HR). Excluded studies were: (1) duplicate articles or full text not available; (2) non-population studies: genetic or cellular studies, animal experiments, etc.; (3) special populations: pregnant women, newborns, psychiatric patients, coal miners, etc.; and (4) lack of key variables or abnormal values. A total of 12,998 papers were retrieved in the final stage, and the quality was evaluated using the Newcastle‒Ottawa‒Scale (NOS) [22]. We included literature with NOS scores up to 5 and above.

### Data extraction and processing

The following elements were extracted (1) basic information: research date, places, sample size, sex, age, study type and published journals; (2) outcomes: lung cancer; (3) smoking exposure: smoking status (smoking or not, current or past smoking), cigarettes smoked per day, pack-years and years of smoking or cessation; and (4) effect values and confidence intervals, models, and correction factors.

The data processing stage included (1) deleting the former results from the same study and (2) removing abnormal data, such as point values less than the lower limit of the confidence interval and RR less than 1 for lung cancer caused by smoking. For the pack-years of smokers, quit-years of quitters and RRs for lung cancer death or prevalence, the median of the upper and lower limits of the interval was taken for closed intervals and 1.2 times the lower limit of the interval was taken for open intervals [23–25].

### Dose‒response RR models

Referring to previous studies [26], we assumed that the risk of lung cancer from smoking was similar in both sexes, and we did not distinguish the prevalence and mortality of lung cancer, as in GBD studies. This study focused on fitting the dose‒response relationship function RR(x) between pack-years of smoking and the RR of lung cancer. The following 10 linear and nonlinear candidate models were built to fit the dose‒response relationship.

The first and second alternative models assumed linear relationships in RR, and these two models were modified from Cohen et al. [27].

Model 1 is a piecewise linear function, assuming a linear relationship between pack-years and RR, with a cutoff value of 30 pack-years, as 30 pack-years is mostly used as the maximum dose in current literature studies, assuming that RR is fixed after pack-years reach 30. The expressions are $x<30,y=\alpha +\gamma \times x; x\ge 30,y=\alpha +\gamma \times 30$.

Model 2 is also a piecewise linear function. The difference from Model 1 is that the boundary value is taken as 45 package years, and the expressions are $x<45,y=\alpha +\gamma \times x; x\ge 45,y=\alpha +\gamma \times 45$.

The third and fourth alternative models assumed power relationships in RR, and were modified from Cohen et al. [27] and Ostro et al. [28].

Model 3 assumes that RR grows exponentially as a power with the increase in pack years, and the power function expression is $y={\{\left(1+x\right)\}}^{\gamma }$.

Model 4 is a power function, and the expression is $y={\{\left[\frac{\alpha +x}{\alpha }\right]\}}^{\gamma }$.

Model 5 is also a power function with the expression $y=1+\alpha \times {x}^{\gamma }$.

The sixth, seventh, and eighth models were modified from Pope et al. in 2009 and in 2011 [29, 30].

Model 6 assumes that RR grows exponentially with the increase in pack years, and the exponential function expression is $y=\alpha -\beta \times {\gamma }^{x}$.

Model 7 is also an exponential function with the expression $y=\alpha \times {\beta }^{x}$.

Model 8 is also an exponential function, and the expression is $y=\alpha +\beta ({\mathrm{e}}^{\{\frac{x+\gamma }{\theta }\}})$.

The last two models were built based on the integrated exposure response (IER) function [31].

Model 9 is the IER function, which slows down the overall growth rate by increasing the parameter -γ, especially when x takes higher values, with the expression $y=1+\alpha (1-{e}^{-\gamma \times x})$ *.*

Model 10 is the IER function, which further uses β to restrict x. The expression of the function is $y=1+\alpha (1-{e}^{-\gamma \times {x}^{\beta }})$.

In the above expression, x is the pack-years, y is the RR, and α, β, γ and θ are the parameters to be adjusted in the models. During the fitting process, the parameter range was initially limited by referring to the model established by GBD in the air pollution study [31], the model parameters were limited according to the actual smoking exposure range, and the final values of the parameters were obtained after several iterations. The degree of model fit was judged according to the Akaike information criterion (AIC) and Bayesian information criterion (BIC) values. The model with the smallest AIC and BIC values was selected as the best-fit model.

For former smokers, we established the RR(y) function, borrowing ideas from GBD [32], to avoid overestimating the RR of quitters with a lighter smoking history. The combined dichotomous RR from meta-analysis was used as the RR corresponding to the starting point (0 years of quitting), namely, the RR at the time of quitting is equivalent to the average RR of current smokers in the same population. Then, the final quit function was obtained by correcting the RR [32].

### Comparison of dose‒response RR models with GBD

The RRs fitted in this study were contrasted with the RRs fitted in accordance with each of the 10 PYs and QYs reported in the Annex of the GBD 2019 study on the dose‒response relationship between smoking and lung cancer [17].

Data analyses were performed by SAS 9.4, and nonlinear model fitting was carried out by using the "Proc nlin" module.

## Results

### Characteristics of the extracted data

A total of 12 published studies on the dose‒response relationship between smoking exposure and lung cancer risk in the Chinese population were included in this study, including 10 case‒control studies and 2 cohort studies (Table 1). The flowchart of the screening process is shown in Figure S1. The study period spanned 31 years, from 1984 to 2015, with a lag time of approximately 5 years from study conduct to publication; the study region was predominantly central-eastern; the sample size fluctuated widely, from 601 to 10,890 cases; the population aged 55 years and older was predominant; the endpoint events were predominantly clinically diagnosed lung cancer (83.33%); and the mean NOS score of the included studies was 6.5. Studies related to smoking and lung cancer were predominant, including 10 studies of pack-years and 2 studies of quit-years. The interval classification of pack-years included 20 pack-years (1–19, 20–39 and ≥ 40 pack-years), 25 pack-years (1–24, ≥ 25 pack-years), 30 pack-years (1–29, ≥ 30 pack-years), 35 pack-years (1–34, ≥ 35 pack-years) and unequal intervals (1–15, 15–19, ≥ 20 pack-years); years of abstinence were divided into intervals of 5 years of abstinence (1–4, 5–9, ≥ 10 quit-years; and 1–4, ≥ 5 quit-years).

**Table 1:** Summary of the included dose‒response relationship studies

Smoking	Number	Publish Year	Sample size	Age	Date	Endpoint	PY/QY interval^a^
Current	10	1996–2021	601–10,890	50 +	1986–2015	prevalence, mortality	5,15,20,25,30
Past	2	1998, 2014	1677–2899	35 +	1984–2010		5

^a^ *PY* Pack-years, *QY* Quit-years

### Quality evaluation of included data

The included dose‒response relationship studies were predominantly of moderate-to-high quality (75%); with 2 cohort studies scoring up to 9. The low score items of NOS in case‒control studies were case representativeness (30%), exposure ascertainment or blinding (50%), and non-response rate (0%), and all the other items were high scored with more than 70% (Table 2).

**Table 2:** NOS scores of each item in the included studies

General items	Case–control study (*n* = 10)		Cohort study (*n* = 2)	
	Detailed items	Score (%)	Detailed items	Score (%)
Selection	1) Is the Case Definition Adequate?	8 (80.00)	1) Representativeness of the Exposed Cohort	2 (100.00)
	2) Representativeness of the Cases	3 (30.00)	2) Representativeness of the Non-Exposed Cohort	2 (100.00)
	3) Selection of Controls	7 (70.00)	3) Ascertainment of Exposure	2 (100.00)
	4) Definition of Controls	8 (80.00)	4) Demonstration That Outcome of Interest Was Not Present at Start of Study	2 (100.00)
Comparability	Comparability of Cases and Controls on the Basis of the Design or Analysis	19 (95.00)	Comparability of Cohorts on the Basis of the Design or Analysis	4 (100.00)
Exposure	1) Ascertainment of Exposure or Blinding	5 (50.00)	1) Assessment of Outcome	2 (100.00)
	2) Same Method of Ascertainment for Cases and Controls	10 (100.00)	2) Was Follow-Up Long Enough for Outcomes to Occur	2 (100.00)
	3) Non-Response Rate	0 (0.00)	3) Adequacy of Follow Up of Cohorts	2 (100.00)
Total score		60 (66.67)		18 (100.00)

### RR function of the dose‒response relationship

**Fig. 1:**
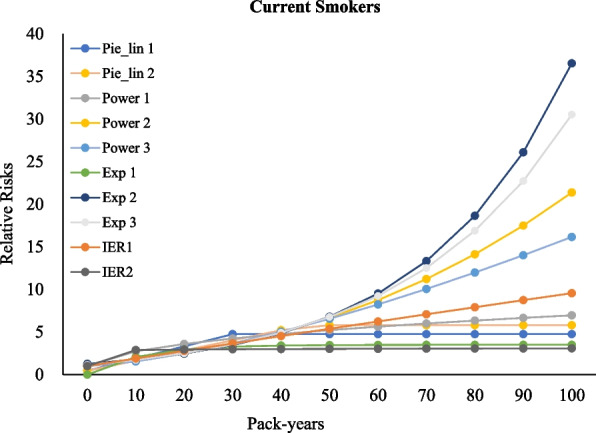
The risk functions of pack-years and relative risk of lung cancer among smokers. Pie_lin: piecewise linear; Exp: exponential; IER: integrated exposure response

**Fig. 2:**
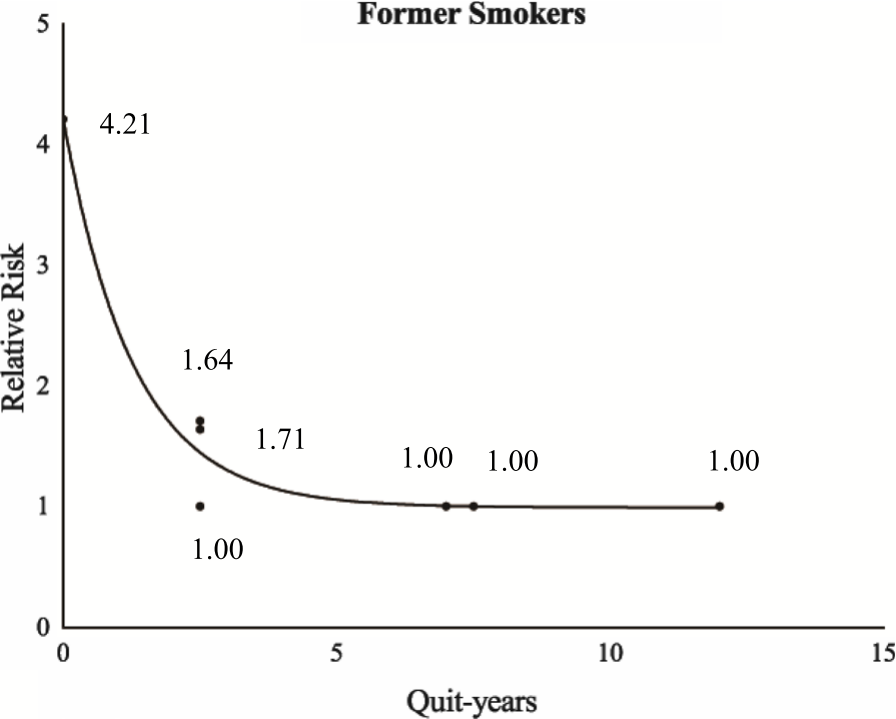
The risk function of quit-years and lung cancer among former smokers

Table 3 shows the final functions and AIC and BIC values for the 10 models, and Fig. 1 shows the different functional forms of pack-years and RR. The RRs were within 10 when pack-years were between 0 and 60 pack-years, and RR up to more than 10 only in the power function and exponential function when pack-years were over 60, whereas RRs among all the other functions were less than 10. Among the 10 models, two piecewise linear functions were up to threshold values when pack-years were equal to 30 and 50, and the corresponding RRs were 4.51 and 5.81; the RRs of the three power functions were 6.96, 21.38, and 16.16 when pack-years were up to 100, respectively; the RRs of the exponential functions were 3.50, 36.54, and 30.52 with 100 pack-years. The RRs of the two IER models were 9.56 and 3.08. In a comprehensive comparison of 10 models, exponential Model 2 rose most sharply with the largest RR values, whereas the piecewise linear functions had a lower RR. The IER models had a relatively stable increase with middle RR values. According to the AIC and BIC, IER Model 1 achieved the best fit (Table 3). The risk function of quit-years and RR was $y=3.2173\times {e}^{(-x/1.2803)}+0.9928$. The RR dropped to 1 when cessation years were up to 7 years and above (Fig. 2).

**Table 3:** Dose‒response relationship models of pack years exposure and relative risks of lung cancer caused by smoking

Models	Functions	AIC	BIC
Piecewise linear model 1	$0<x\le 30,y=0.45+0.1434\times x$ $x>30, y=4.75$	4.20	32.58
Piecewise linear model 2	$0<x\le 45, y=0.45+0.1192\times x$ $x>45,y=5.81$	-128.00	-165.00
Power function model 1	$y={(1+x)}^{0.4205}$	-69.33	-106.11
Power function model 2	$y=({(58.43+x)/58.43)}^{3.07}$	-56.30	-93.11
Power function model 3	$y=1+0.0194\times {x}^{1.4465}$	-88.68	-124.45
Exponential model 1	$y=3.5-3.704\times {0.909}^{x}$	-23.09	-59.87
Exponential model 2	$y=1.2655\times {1.0342}^{x}$	-35.13	-71.90
Exponential model 3	$y=-0.6+0.98\times {\mathrm{e}}^{(x+20)/34.7}$	-45.10	-81.88
IER model 1	$y=1+89.95\times (1-{\mathrm{e}}^{-0.001\times x}$	-311.12	-347.89
IER model 2	$y=1+7.2324\times (1-{\mathrm{e}}^{-0.2697\times {x}^{0.0497}})$	-285.40	-321.17

*AIC* Akaike information criterion, *BIC* Bayesian Information Criteria, *IER* Integrated-exposure–response

### Comparison of dose‒response relationship RRs

**Fig. 3:**
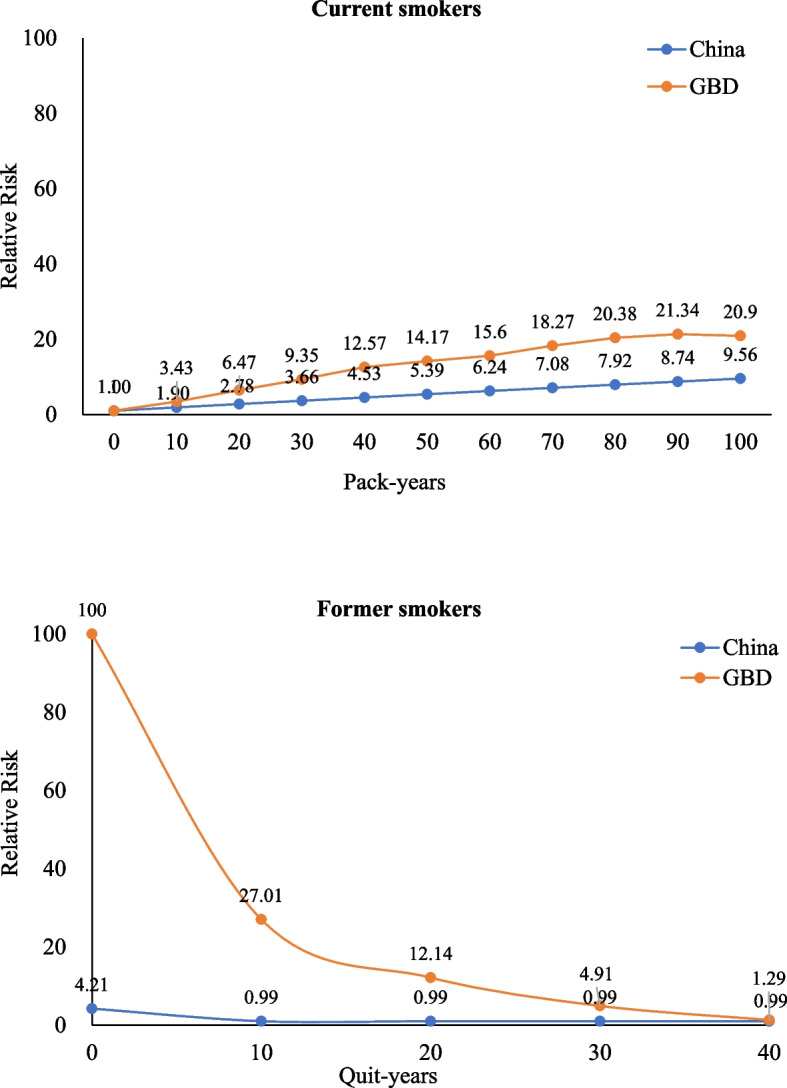
Comparison of dose‒response relative risk. 3–1 Dose‒response relative risk of current smokers; 3–2 Dose‒response relative risk of former smokers

In GBD studies, researchers used a global RR model to present the dose‒response relationship between smoking and lung cancer. In 2019, the RR stabilized when pack-years reached 90, with a maximum RR value of 21.34 [17]. The dose‒response relationship RRs of pack-years and quit-years estimated in this study were far below the global level (Fig. 3).

## Discussion

### Main findings

Among smokers, the RR for lung cancer death increased with pack-years. The IER model presented the best fit for the dose‒response relationship between pack-years of smoking and RR, with a corresponding RR of 9.56 when pack-years reached 100. Among quitters, the RR decreased to approximately 1 when individuals quit smoking after 7 years. The RR values calculated by the dose‒response relationship method in our study were much lower than the global RR values estimated in GBD studies.

### The dose‒response RR is more representative of the risk of smoking

Individuals in the general population have previously been classified into smokers and nonsmokers with dichotomous RR values; however, the smoking habits, as well as the risk of lung cancer, vary among different smokers. The pack-year indicator integrates smoking in two dimensions: time period and amount of smoking [33]. Usually, heavy smokers with a long smoking history have a much higher risk of developing lung cancer than light smokers with a short history. The pooled dichotomous RRs of lung cancer death based on the same database were 3.26 (95% CI: 2.79–3.82) and 3.18 (2.78–3.63) for males and females, respectively [34]. By comparison, dose‒response relationship studies have shown that the RR of lung cancer among smokers increases with pack-years, especially after 40 pack-years, with an RR reaching 5, and the risk decreases significantly with quit-years, with the RR close to 1 after 7 years of abstinence. Therefore, pack-years could work as a clear indicator for quantifying the cumulative intensity of smoking among current smokers, and quit-years could provide more reasonable evidence for the risk estimation of former smokers.

## Discussion of methods

The IER model was initially applied in the environmental field to present the effects of air pollution on health outcomes and is especially applicable for high exposure levels. The IER model exhibits a "ceiling effect", with a steep curve at low exposures that smooths out at high exposures; as epidemiological evidence accumulates, the model can be updated based on a priori information [35]. Therefore, the IER model was able to depict the dose‒response relationship between various cumulative exposure dosages. In addition, statistically, the IER model also fitted best, with the smallest AIC and BIC values among the models developed in this study.

Due to limited evidence, we made the following hypotheses: (1) an equal OR, RR and HR: since more case‒control studies were included in this study, the effect values were dominated by ORs, and to avoid more errors, this study combined the effect values directly without corrections; (2) an equal RR for prevalence and mortality in lung cancer: this assumption was consistent with the GBD framework [18], and current evidence does not support a significant difference between the two outcomes; and (3) equal smoking-attributable risk of lung cancer for males and females due to limited evidence and no difference in male and female dichotomous RRs.

### The RR comparison

There are various risk factors for lung cancer in China. Indoor air pollution caused by unique Chinese cooking styles and outdoor air pollution induced by environmental damage are strong competitors for the effects of smoking [36]; therefore, the RR of smoking-caused lung cancer in the Chinese population has been known to differ significantly from that in Western countries. In this study, the dose‒response relationship-based RR of smoking-induced lung cancer mortality in the Chinese population was significantly lower than the global estimates calculated by GBD 2019. The RR of smoking-induced lung cancer in the Chinese population was less than 5, whereas the RR reached 15 in GBD studies when smokers smoked less than 50 pack-years. The underlying mechanism could be: (1) smoky coal attenuating the association between smoking and lung cancer risk [9] and (2) interactions between carcinogens in fossil fuel and tobacco smoke.

Since 2017, GBD has adopted globally uniform RRs. In the dose‒response relationship, the evidence from the included studies was mainly from developed countries, and only 1 case‒control study conducted in the Chinese population in 1996 was included in GBD 2017 [37]; therefore, it seems unreasonable to estimate PAFs in the Chinese population by using the RR from global studies mostly performed in developed countries.

### Importance of this study

This is the first study to explore the dose‒response relationship between smoking and the risk of lung cancer development or death in the Chinese population based on pack-years for smokers and years since cessation for quitters. The results showed some differences between the RRs between smoking and the risk of lung cancer in the Chinese population and those estimated at the global level in the GBD, suggesting that the GBD should still estimate the RRs of smoking and resulting disease in the Chinese population separately when estimating the risk of death from lung cancer due to smoking. Given the limited evidence, high-quality, large prospective cohort-based studies in the Chinese population should be strongly encouraged in the future to further explore the dose‒response relationships of pack-years and quit-years with the risk of smoking-attributable disease to more accurately estimate the burden of smoking-attributable disease.

### Limitations

This study had the following shortcomings: (1) this study was limited by the number of studies and the quality of studies, and ultimately included fewer studies, especially those related to smoking cessation; therefore, this study somewhat compensated for the limitations of insufficient data by building multiple models to portray the dose‒response relationships in different scenarios; (2) this study could not distinguish between different sexes because of limited evidence; given that the dichotomous results showed no significant differences between men and women, it would not cause large bias in this study; (3) this study only focused on active smoking and did not take passive smoking into account, which may slightly underestimate the risk due to smoking; and (4) the definition of smoking varied from study to study (table S2), inducing certain heterogeneity; considering that most studies used pack-years or lifelong smokers smoked more than 100 cigarettes to define ever smokers, this bias was acceptable.

## Supplementary Information

**Additional file 1.**

**Additional file 2.**

## Data Availability

The datasets used and/or analysed during the current study available from the corresponding author on reasonable request.
