# Supplementary material for: Dose–response relationship between active smoking and lung cancer mortality/prevalence in the Chinese population: a meta-analysis
Source: BMC Public Health. 2023 Apr 24;23:747. doi: 10.1186/s12889-023-15529-7 (PMC10124028; doi:10.1186/s12889-023-15529-7)
Supplement: Supplementary file 2 — Additional file 2. [file 12889_2023_15529_MOESM2_ESM.docx]

# Supplementary Materials

Supplementary table 1 Characteristics of included dose-response relationship studies

| First Author | Year | Region | Gender | Sample size | Age, year | Date | Endpoint | PY/QY | NOS |
| --- | --- | --- | --- | --- | --- | --- | --- | --- | --- |
|  |  |  |  |  |  |  |  | interval^*^ |  |
| Yi-xiong Lei | 1996 | Guangdong | male | 1,384 |  | 1986 | prevalence | 20 | 5 |
| Moira Chan-Yeung | 2003 | Hong Kong | male | 662 | 55–57 | 1999–2001 | prevalence | 20 | 7 |
| Zhiqiang Liu | 2015 | Fujian | male | 1,878 |  | 2006–2013 | prevalence | 20 | 6 |
| Jinman Zhuang | 2021 | Fujian | male | 3,244 | 59 | 2006–2015 | prevalence | 20 | 7 |
| Wuwilliams, A. H. | 1990 | Liaoning | female | 1,923 | 55 | 1985–1987 | prevalence | 15，5 | 6 |
| X.-R. Wang | 2009 | Hong Kong | female | 601 | 65 | 2002–2004 | prevalence | 25 | 7 |
| Yuk-Lan Chiu | 2010 | Hong Kong | female | 601 | 65 | 2002–2004 | prevalence | 25 | 6 |
| Zi-Yi Jin | 2013 | Jiangsu | both | 5,967 | 63–64 | 2003–2010 | prevalence | 30 | 5 |
| Jin-Kou Zhao | 2017 | Jiangsu | both | 10,890 | 64 | 2003–2010 | prevalence | 20 | 6 |
| YU-TANG GAO | 1988 | Shanghai | male, female | 2,899 | 35–69 | 1984–1986 | prevalence | 5^**^ | 5 |
| He, Y.^#^ | 2014 | Shaanxi | both | 1,494 | 51.5–87.8 | 1994–2011 | mortality | 35 | 9 |
| Chang, L. C. ^#^ | 2014 | Taiwan | both | 1,677 | ≥65 | 2001–2010 | mortality | 5^**^ | 9 |

Note: ^*^PY: pack-year; ^**^QY: quit-year; ^#^ cohort study

Supplementary table 2 Smoking definition of included dose-response relationship studies

| Studies | Status | Definition |
| --- | --- | --- |
| Yi-xiong Lei, 1996 | Ever | Not specified. |
| Moira Chan-Yeung, 2003 | Ever | Ever-smoker: one who had smoked at least one cigarette a day, pipe, water pipes, cigars, and/or hand rolled cigarettes, for 1 year or more. |
| Zhiqiang Liu, 2015 | Ever | Smoking was defined who had smoked more than 100 cigarettes in their life. |
| Jinman Zhuang, 2021 | Ever | Smoking was defined if participants reported to have smoked a total of at least 100 cigarettes in their lifetime. |
| Wuwilliams, A. H., 1990 | Ever | Not specified. |
| X.-R. Wang, 2009 | Ever | An ever smoker was defined as one who had ever smoked >20 packs of cigarettes in lifetime, more than one cigarette a day, or more than one cigar a week for 1 year. |
| Yuk-Lan Chiu, 2010 | Ever | Smoker was defined as one who had ever smoked more than 20 packs of cigarettes in lifetime, or more than one cigarette a day, or more than one cigar a week for 1 year. |
| Zi-Yi Jin, 2013 | Ever | Not specified. |
| Jin-Kou Zhao, 2017 | Ever | Ever smokers were defined as individuals who had smoked at least 100 cigarettes in their lifetimes. |
| He, Y., 2014 | Current | Current smokers were persons who were current smokers at either the 1976 baseline or the 1994 follow-up. |
| YU-TANG GAO, 1988 | Former | Not specified. |
| Chang, L. C.， 2014 | Former | Former smokers were those who had smoked more than 100 cigarettes in their life time but did not smoke in the past month. |

Supplementary Figure

Figure S1 Flowchart
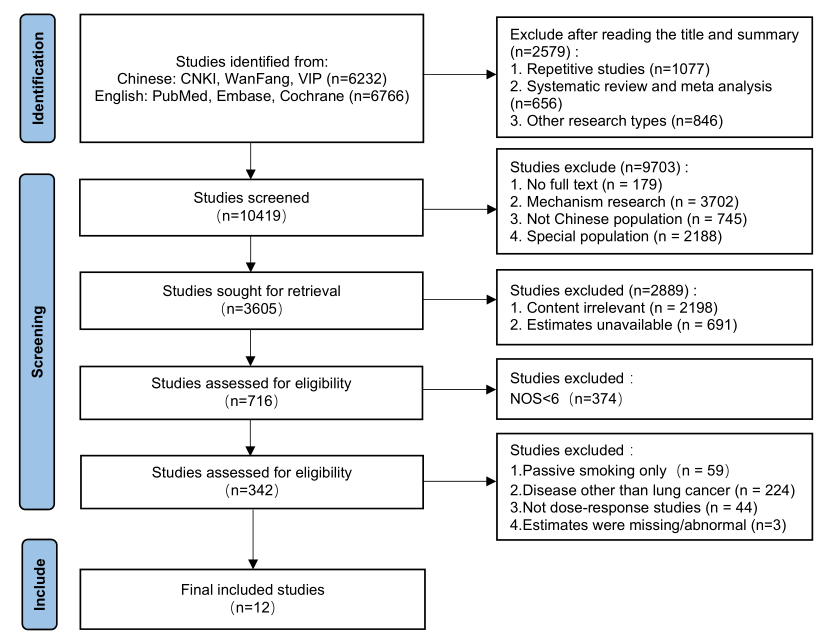
 of the literature screening process
